# Acute exposure to carbon monoxide inhalation and/or hot water immersion transiently increases erythropoietin in females but not in males

**DOI:** 10.1113/EP091923

**Published:** 2024-08-15

**Authors:** Kaitlyn G. DiMarco, Christopher L. Chapman, Natasha E. Weiser, Emma R. Matsell, Kathryn M. Lucernoni, Samantha Chacon, Margaret M. B. Grivette, John R. Halliwill, Andrew T. Lovering, Christopher T. Minson

**Affiliations:** ^1^ Department of Human Physiology University of Oregon Eugene Oregon USA

**Keywords:** carbon monoxide, erythropoietin, heat stress, hot water immersion, sex differences

## Abstract

The use of acute carbon monoxide inhalation (COi) and hot water immersion (HWI) are of growing interest as interventions to stimulate erythropoietin (EPO) production. However, whether EPO production is further augmented when combining these stressors and whether there are sex differences in this response are poorly understood. Therefore, we measured circulating EPO concentration in response to acute COi and HWI independently and in combination and determined whether the responses were altered by sex. Participants completed three study visits—COi, HWI, and combined COi and HWI—separated by 1 week in a randomized, balanced, crossover design. Renal blood velocity was measured during all interventions, and carboxyhaemoglobin was measured during and after COi. Serum samples were analysed every hour for 6 h post‐intervention for EPO concentration. HWI decreased renal blood velocity (46.2 cm/s to 36.2 cm/s) (*P *< 0.0001), and COi increased carboxyhaemoglobin (1.5%–12.8%) (*P *< 0.0001) without changing renal blood velocity (46.4–45.2 cm/s) (*P* = 0.4456). All three interventions increased peak EPO concentration from baseline (COi: 6.02–9.74 mIU/mL; HWI: 6.80–11.10 mIU/mL; COi + HWI: 6.71–10.91 mIU/mL) (*P* = 0.0048) and to the same extent (*P* = 0.3505). On average, females increased EPO while males did not in response to COi (females: 6.17 mIU/mL; males: 1.27 mIU/mL) (*P* = 0.0010), HWI (females: 6.47 mIU/mL; males: 2.14 mIU/mL) (*P* = 0.0104), and COi and HWI (females: 6.65 mIU/mL; males: 1.76 mIU/mL) (*P* = 0.0256). These data emphasize that combining these interventions does not augment EPO secretion and that these interventions may work better in females.

## Abstract (highlights)

- **What is the central question of the study?**
  Do acute heat stress and carbon monoxide inhalation alone and in combination increase erythropoietin, and are there sex differences in erythropoietin?
- **What is the main finding and its importance?**
  All interventions increased erythropoietin, but combining the two interventions did not further augment erythropoietin. While erythropoietin increased in females in response to all interventions, there was greater erythropoietin variability in males and therefore, on average, males did not increase erythropoietin. This suggests that combining heat and hypoxic stressors does not further augment erythropoietin concentrations but these interventions may have greater efficacy in females.

## 1 INTRODUCTION

When oxygen delivery to the kidneys is reduced, secretion of erythropoietin (EPO) from EPO‐producing interstitial peritubular cells around the proximal tubules of the renal cortex, and to a lesser extent the outer medulla, markedly increases (Jacobson et al., 1957; Lacombe et al., 1988). The result is an elevation of circulating EPO that returns to baseline once renal oxygen delivery is restored (Abbrecht & Littell, 1972; Berglund et al., 2002; Faura et al., 1969; Ge et al., 2002; Jelkmann, 2011). Renal oxygen delivery comprises the combined effect of renal blood flow and arterial oxygen content ($C_{aO_{2}}$). Accordingly, acute carbon monoxide inhalation (COi) and hot water immersion (HWI) are potential interventions to increase EPO concentration because of their likelihood of selectively reducing $C_{aO_{2}}$ and renal blood flow, respectively.

Unlike chronic heat stress, which results in plasma volume expansion and subsequent alterations in $C_{aO_{2}}$ that may be at least partially responsible for chronic haematological adaptations to heat (Nybo et al., 2022), acute heat stress most likely stimulates EPO production by reducing renal blood flow. While heat can induce a rightward shift in the oxyhaemoglobin dissociation curve, small increases in core temperature at sea level do not elicit drastic reductions in arterial oxygen saturation ($S_{aO_{2}}$) and therefore $C_{aO_{2}}$ (Kelman, 1966). Rather, acute heat stress causes renal vasoconstriction via increased renal sympathetic nerve activity, which reduces renal perfusion to support cutaneous vasodilatation (Rowell et al., 1970). Notably, there is some evidence indicating that blood flow in the renal cortical region, where EPO is produced, is most affected by passive heat stress. Hyperthermia caused a heterogenous redistribution of intrarenal blood flow in dogs, whereby marked reductions in cortical renal blood flow occurred with a relative maintenance of medullary renal blood flow (Miyamoto, 1994). Importantly, reductions in renal blood flow do not appear to be met with a compensatory increase in renal oxygen extraction and therefore can elicit renal hypoxaemia (Hess et al., 2022; Kim et al., 2021; Levy, 1960). Thus, it is biologically plausible that passive heat stress in humans may stimulate EPO production by reducing renal cortical blood flow and subsequently eliciting renal cortical hypoxaemia, although this has never been confirmed in humans.

Alternatively, given that EPO production is responsive to reduced $C_{aO_{2}}$ independent of arterial oxygen tension (Montero & Lundby, 2019), COi most likely stimulates EPO production by reducing functional $S_{aO_{2}}$ without reducing arterial oxygen tension. There is limited evidence indicating whether COi alters renal blood flow in healthy adults. In one study, renal plasma flow appeared to be acutely increased 6–12 h following two doses of COi administration (average HbCO ranged between 11.5% and 13.6%), but there was large variability in the renal plasma flow response and statistical analyses were not performed due to the low number of participants (Pauli et al., 1968). In other settings, COi has been proposed to limit acute renal injury, and endogenously produced renal CO may be protective against excessive renal vasoconstriction (Csongradi et al., 2012; Kwong et al., 2022). Additionally, lower doses of COi (HbCO of 8.3%) are insufficient to increase sympathetic nerve activity (Hausberg & Somers, 1997) and subsequent renal vasoconstriction while higher doses of COi (HbCO of 23–24%) do increase sympathetic nerve activity (Hanada et al., 2003). Therefore, the effect that COi independently may have on renal blood flow is unknown.

While previous studies have shown acute COi can stimulate EPO production (Montero & Lundby, 2019; Schmidt et al., 2020; Wang et al., 2019), whether the imposition of heat stress, which reduces renal blood flow and may counteract any potential vasodilatory effect of COi, further increases EPO requires further investigation. Additionally, a significant knowledge gap exists regarding the characterization of these responses in females, as the few studies that have investigated EPO concentration in response to acute COi have been conducted only in males (Montero & Lundby, 2019; Schmidt et al., 2020; Wang et al., 2019). However, there is no evidence that there are sex differences in EPO concentration, as baseline reference ranges for EPO concentration in females and males are the same (Beverborg et al., 2015).

Therefore, the purposes of this study were threefold. Primarily, we sought to determine whether COi and HWI would increase EPO concentration and whether combining HWI and COi (COi + HWI) would augment EPO secretion. Secondly, we sought to characterize the stimulus for EPO production in response to each intervention, and lastly, we sought to determine whether there were any sex differences in these responses. Specifically, we hypothesized that (1) all three interventions would increase EPO concentration but COi + HWI would augment increases in EPO concentration, (2) COi would selectively reduce $C_{aO_{2}}$ without reducing renal blood flow while HWI would reduce renal blood flow, and (3) there would be no sex differences in the change in EPO concentrations following all three interventions.

## 2 METHODS

### 2.1 Ethical approval and participant population

Twenty‐one individuals volunteered to participate in this study after being advised both verbally and in writing about the nature of the experiments. Participants signed an informed consent form to participate in the study approved by the University of Oregon Research Compliance Services (protocol STUDY00000189). All studies were performed in accordance with the standards set forth by the *Declaration of Helsinki* except for registration in a database. Of these 21 volunteers, four participants were lost to follow‐up. Of the remaining 17 participants that completed data collection, one participant had EPO concentrations below limits of detection for all time points during all interventions and was, therefore, excluded from analyses (see ‘EPO analysis’ below). Therefore, data are presented for 16 participants (*n* = 8 females and *n* = 8 males).

### 2.2 Study design and instrumentation

**FIGURE 1:**
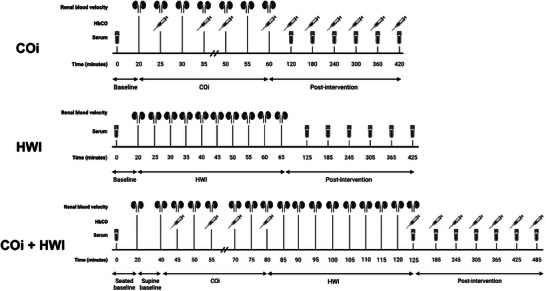
Overview of the study design. Participants completed three interventions in a randomized, balanced, crossover design. Data collected included renal blood velocity (kidney icon) via Doppler ultrasound, carboxyhaemoglobin (HbCO; syringe icon) and serum samples (blood tube icon). Serum was used to quantify EPO concentration. See ‘Methods’ for a complete description.

Participants first completed a screening visit, during which a blood draw for measuring iron status (iron, ferritin and transferrin) and anthropometric data (height, weight and age) were obtained. Additionally, the international physical activity questionnaire was administered to quantify normal weekly physical activity (Craig et al., 2003). After screening, participants completed three experimental visits (COi, HWI, and COi + HWI) in a balanced and randomized crossover design, illustrated in Figure 1. A time control visit was not performed because EPO does not increase over time following breathing room air (Montero & Lundby, 2019). Given the acute nature of the study, the visit was separated by at least 1 week to avoid any potential influencing effects of the interventions (e.g., heat acclimation). All participants started the study visits in the morning between 06.30 and 08.30 h.

Upon arrival on all study visits, participants provided a urine sample for measurement of urine specific gravity. All participants had urine specific gravity <1.020 or were asked to consume 5 mL/kg body weight of water if ≥1.020. Afterwards, participants were instrumented with an intravenous (i.v.) catheter for blood draws. In addition, participants were instrumented with a blood pressure cuff to measure brachial artery blood pressure during the interventions (SunTech Tango M2 Stress Test Monitor, Morrisville, NC, USA). On the days that involved HWI, participants were instrumented with either a rectal temperature probe (ZOLL Medical Corporation YSI Reusable rectal temperature probe, Chelmsford, MA, USA; *n* = 8) or a core temperature pill (HQInc CorTemp Sensor, Palmetto, FL, USA; *n* = 8) for monitoring core temperature. Participants ingested the pill approximately 10 h prior to their study visit. Nude weight was measured before and after HWI to estimate whole‐body sweat loss. After instrumentation, baseline measurements were made (see ‘Measurements’ below), and then participants completed the assigned intervention. Subsequently, participants had blood drawn from the i.v. catheter once every hour for 6 h post‐intervention. On the COi + HWI visit, participants had one additional post‐intervention blood draw immediately after HWI (0 h time point), which corresponded to 1 h after COi. Participants were permitted to leave the lab but were instructed not to exercise between blood draws.

### 2.3 Interventions

For the COi visit, participants completed duplicate haemoglobin (Hb) mass measurements via the 10‐min optimized CO rebreathe technique (Siebenmann et al., 2017). This technique was chosen because our labs have experience consistently achieving a peak HbCO of 10–15% in this participant population, and it would allow us to conduct the study while minimizing participant visits. Participants began by resting supine for 20 min for normalization of plasma volume shifts due to gravity (Hagan et al., 1978; Keiser et al., 2013). After 20 min of supine rest, we obtained baseline measurements (see ‘Measurements’ below). Afterwards, participants breathed 100% O_2_ for 4 min followed by rebreathing a small bolus of CO in 100% O_2_ for 10 min. The volume of CO was determined based on participant weight and was different between sexes to account for sex differences in Hb mass (0.8 mL/kg body weight for women and 1.0 mL/kg body weight for men) (Siebenmann et al., 2017). After the first Hb mass test, participants completed a duplicate Hb mass test.

For the HWI visit, participants sat upright in a hot tub heated to 40°C for 45 min. We specifically chose HWI instead of another heating modality because of its ability to quickly and effectively increase core temperature (Chapman et al., 2021), and passive heat stress in dogs was sufficient to reduce renal cortical blood flow (Miyamoto, 1994). Prior to entering the hot tub, participants rested in a seated upright position for 20 min in the laboratory (temperature = 23.0 ± 0.8°C; humidity = 37.3 ± 10.3%). After 20 min of upright rest, we obtained baseline measurements (see ‘Measurements’ below). While in the hot tub, we sought to increase the core temperature to 38.5°C with water immersion up to the sternum. In the event that a core temperature of 38.5°C was reached (*n* = 4), water immersion up to the waist was performed and a fan was turned on in an attempt to clamp the core temperature at 38.5°C to improve participants’ thermal perception at this elevated core temperature. All participants were given 2 mL of water per kg of body weight after 25 min in the hot tub.

On the COi + HWI visit, participants completed the above interventions back‐to‐back. COi occurred first, followed by HWI, to ensure a quick transition between interventions. We have previously shown that following passive heat stress, which reduces renal artery blood velocity, renal perfusion is restored when skin temperature returns to baseline despite core temperature remaining elevated (Chapman et al., 2019). Therefore, it was necessary that HWI always followed the COi in the HWI + COi visit to ensure that a sufficient combined stimulus was provided (i.e., renal blood flow would otherwise be restored to baseline by the time the effects of COi are observed if HWI was administered first). Baseline data were obtained prior to either intervention in both an upright position and supine. This allowed us to compare measurements during the intervention to the baseline data in the same body position.

### 2.4 Measurements

#### 2.4.1 Renal blood velocity, mean arterial pressure and renal vascular resistance

Renal artery blood velocity was measured via Doppler ultrasound before (baseline measurements) and during every intervention as previously described (Chapman et al., 2023, 2020). Renal artery blood velocity is considered a reliable proxy for renal artery blood flow because of evidence from invasive human studies demonstrating that pharmacologically induced renal vasoconstriction causes decreases in renal artery blood velocity with no changes in renal artery diameter (Rocha et al., 2023). Thus, vasoconstriction occurs downstream from these large conduit arteries in the renal afferent and/or efferent arterioles within the kidneys (Rocha et al., 2023). Renal blood velocity was measured in the distal segment of the right renal artery using the coronal approach. The same artery, for a given participant, was used throughout the experimental protocol. Baseline measurements were taken in the supine position (Chapman et al., 2023) for the COi intervention, and baseline measurements were taken in the seated position prior to the HWI intervention to replicate the position of participants during the interventions. Before the COi + HWI intervention, baseline measurements were taken in the seated position first and were then taken in the supine position. After both baseline measurements in both body positions were completed, the participant began the COi intervention followed by HWI. The two baseline measurements were deemed necessary so that changes in renal blood velocity could be effectively compared to the same body posture at baseline within a given intervention. During visits involving HWI, a clear surgical sleeve (CIVCO Medical Solutions, Kalona, IA, USA) was used to cover the transducer and allow for underwater renal blood velocity measurements. Renal blood velocity was measured across three consecutive cardiac cycles during which participants were instructed to perform a mid‐exhalation, non‐Valsalva breath hold lasting no more than 10 s. The same sonographer obtained all renal blood velocity measurements (C.L.C.). In addition, the location of the transducer was kept consistent by marking the participant with indelible ink.

During COi, renal blood velocity was measured during the final minute of the 4 min of 100% O_2_, 5 min into the 10‐min CO rebreathe, and during the final minute of the 10‐min CO rebreathe for both tests. While in the hot tub, renal blood velocity was measured every 5 min. Brachial artery blood pressure was measured at the same time points. An index of renal vascular resistance was calculated as mean arterial pressure (MAP)/renal blood velocity.

#### 2.4.2 Venous blood samples

An i.v. catheter was placed into an antecubital vein for obtaining blood draws on all visits, including screening. On the screening visit, participants had 15 mL of blood drawn for analyses of iron, ferritin and transferrin. Blood samples were drawn into serum separator tubes (Becton‐Dickenson, Franklin Lakes, NJ, USA) and kept at room temperature to be analysed within 7 days (QUEST Diagnostics, Secaucus, NJ, USA). Iron was analysed via spectrophotometry, ferritin via immunoassay and transferrin via immunoturbidimetric assay.

On the intervention study visits, prior to the interventions and every hour after the intervention for 6 h, a 15‐mL venous blood sample was drawn from the i.v. catheter into serum separator tubes (Becton‐Dickenson) to measure EPO concentrations. Serum separator tubes were allowed to sit at room temperature for at least 30 min to fully clot, after which they were centrifuged at 1500 *g* for 10 min. Serum was separated and frozen at −80°C until analysed. In addition to serum separator tubes, on the COi and COi + HWI interventions, a 2‐mL venous blood sample was drawn into a heparinized syringe at baseline, post‐Hb mass, and every hour for 6 h post‐intervention. The blood samples collected into heparinized syringes were immediately analysed via co‐oximetry to quantify carboxyhaemoglobin (HbCO). HbCO was used to calculate Hb mass (Siebenmann et al., 2017) and quantify the strength of the COi stimulus.

### 2.5 EPO analysis

EPO was analysed by enzyme‐linked immunosorbent assay (BioLegend LEGENDMAX Human EPO ELISA, San Diego, CA, USA), with an assay sensitivity of 0.25 mIU/mL. Frozen serum samples were fully thawed and refrigerated on ice. Per the manufacturer guidelines, samples were not diluted prior to analysis. All samples were above the limits of detection of the assay except for one participant (noted above in ‘Ethical approval and participant population’) who was excluded from analyses.

### 2.6 Data and statistical analysis

Statistical analyses were performed using GraphPad Prism (version 10.0.3; GraphPad Software, Boston, MA, USA) with *P* < 0.05 considered statistically significant. Data are presented as means ± standard deviation, unless otherwise stated. A one‐way mixed‐effects analysis with Šidák post hoc was performed to compare baseline EPO concentrations from each participants’ first, second, and third interventional visits to confirm no compounding effect of the interventions over time. Similarly, a two‐way mixed effects analysis was performed comparing baseline EPO across visits and sex. HbCO, renal blood velocity, renal vascular resistance, MAP, core temperature, heart rate, and EPO concentrations were analysed over time and between interventions by two‐way mixed effects analyses with Šidák's post‐hoc test.

The change in EPO concentrations was analysed over time and between sex within each interventional visit via a two‐way mixed effects analysis with Šidák's post‐hoc test. To determine if there were sex differences in the screening (height, weight, age, Hb mass, iron, ferritin, transferrin and physical activity hours) and stimulus (peak and ∆ HbCO, minimum and ∆ renal blood velocity, peak and ∆ renal vascular resistance, peak and ∆ core temperature, and peak and ∆ heart rate) variables, Student's unpaired *t*‐test was used.

## 3 RESULTS

### 3.1 Participant characteristics

All participants identified as recreationally active (Table 1) and were free from self‐reported cardiovascular and renal diseases. To the best of their knowledge, no participants reported as being descendants from major high‐altitude populations (Aymaran, Tibetan or Ethiopian); however, one female participant was born in Colorado (elevation of approximately 1675 m). The remaining participants were all born in low‐altitude cities. In addition, none of the participants traveled to altitudes >2000 m for >6 h of time in the month leading up to the study. Participants were not excluded for having iron status parameters outside of the normal limits (*n* = 4 females; *n* = 4 males). Participant anthropometrics, baseline Hb levels, iron status and self‐reported physical activity are presented in Table 1.

**TABLE 1:** Female and male participant screening characteristics.

		Females	Males	*P*‐value
Anthropometrics	Height (cm)	164.0 ± 3.0	178.8 ± 4.7	<0.0001*
	Weight (kg)	69.0 ± 8.7	71.3 ± 6.9	0.5722
	Age (years)	22 ± 2	25 ± 4	0.1089
Haematological	Hb mass (g)	539.6 ± 88.0	841.1 ± 75.8	<0.0001*
Iron status	Iron (µg/dL)	102.5 ± 40.1	138.4 ± 36.3	0.0815
	Ferritin (ng/mL)	25.5 ± 18.1	68.4 ± 47.7	0.0322*
	Transferrin (mg/dL)	306.3 ± 61.5	268.1 ± 14.0	0.1092
Weekly physical activity	Vigorous (h)	4.6 ± 3.0	7.1 ± 4.5	0.2263
	Moderate (h)	3.1 ± 3.7	6.5 ± 14.4	0.5290
	Walking (h)	8.4 ± 6.3	6.6 ± 4.1	0.5080
	Total (h)	16.1 ± 6.4	20.1 ± 16.0	0.5184

*Note*: Data obtained during screening (anthropometrics, iron status, and weekly physical activity) are presented. Hb mass was averaged from the COi and COi + HWI interventions. *n* = 8 females; *n* = 8 males. Females and males were compared using unpaired *t*‐tests. *Significant differences between females and males.

### 3.2 Stimulus for EPO secretion

**FIGURE 2:**
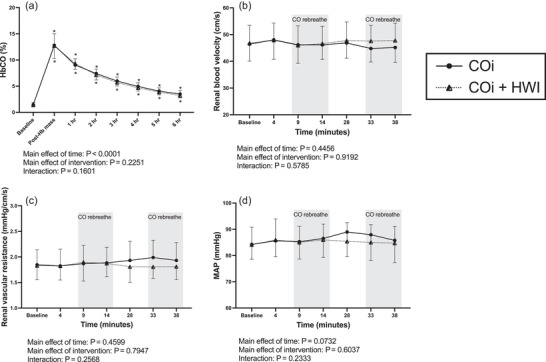
Stimulus for EPO secretion in response to COi. Data are presented as means ± SD. Data were analysed with two‐way mixed‐effects analysis with time and intervention as the two factors. *Significant pairwise comparisons (*P* < 0.05) compared to baseline within an intervention. Main effects of time and intervention, as well as interaction effects, are presented below each panel. COi is represented with filled circles, continuous lines; COi + HWI is represented with half‐filled triangles, dotted lines. For (b–d), shaded bars indicate the measurements taken during CO rebreathe. (a) HbCO on the COi (*n* = 16) and COi + HWI (*n* = 16) visits. (b) Renal blood velocity on the COi (*n* = 14) and COi + HWI (*n* = 14) visits. (c) Renal vascular resistance on the COi (*n* = 14) and COi + HWI (*n* = 14) visits. (d) Mean arterial pressure (MAP) on the COi (*n* = 14) and COi + HWI (*n* = 14) visits.

**FIGURE 3:**
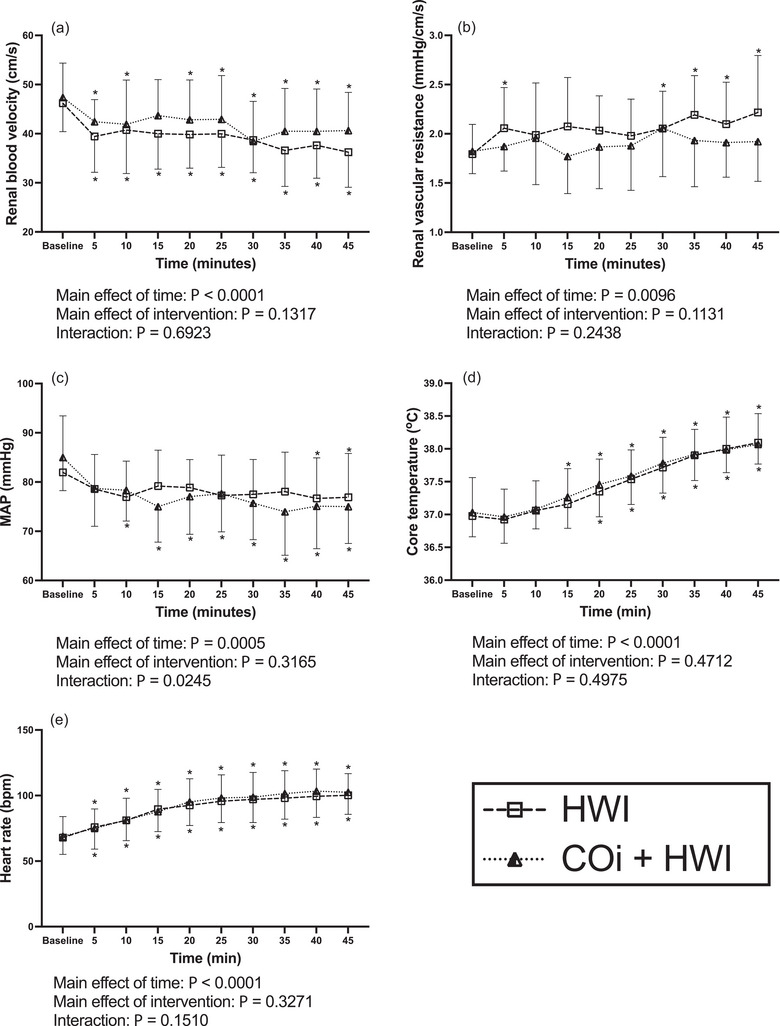
Stimulus for EPO secretion in response to HWI. Data are presented as means ± SD. Data were analysed with two‐way mixed‐effects analysis with time and intervention as the two factors. *Significant pairwise comparisons (*P* < 0.05) compared to baseline within an intervention. Main effects of time and intervention, as well as interaction effects, are presented below each panel. HWI is represented with open squares, dashed lines; COi + HWI is represented with half‐filled triangles, dotted lines. (a) Renal blood velocity on the HWI (*n *= 16) and COi + HWI (*n* = 15) visits. (b) Renal vascular resistance on the HWI (*n* = 16) and COi + HWI (*n* = 15) visits. (c) Mean arterial pressure (MAP) on the HWI (*n* = 16) and COi + HWI (*n* = 15) visits. (d) Core temperature on the HWI (*n* = 16) and COi + HWI (*n* = 16) visits. (e) Heart rate on the HWI (*n* = 16) and COi + HWI (*n* = 16) visits.

HbCO increased following COi and was identical on the COi and COi + HWI visits (Figure 2a). HbCO increased from 1.5 ± 0.5% to 12.8 ± 2.2% on the COi visit and from 1.5 ± 0.4% to 12.7 ± 2.4% on the COi + HWI visit. COi did not change renal blood velocity (Figure 2b), renal vascular resistance (Figure 2c), or MAP (Figure 2d) during either COi or COi + HWI interventions. Conversely, HWI reduced renal blood velocity and MAP and increased renal vascular resistance on both the HWI and COi + HWI visits (Figure 3). Renal blood velocity decreased from 46.2 ± 5.8 to 36.2 ± 7.1 cm/s on the HWI visit and from 47.4 ± 7.0 to 40.6 ± 7.8 cm/s on the COi + HWI visit. Additionally, core temperature and heart rate increased to similar extents during both HWI and COi + HWI interventions (Figure 3d,e).

### 3.3 EPO concentrations in response to COi, HWI and COi + HWI

**FIGURE 4:**
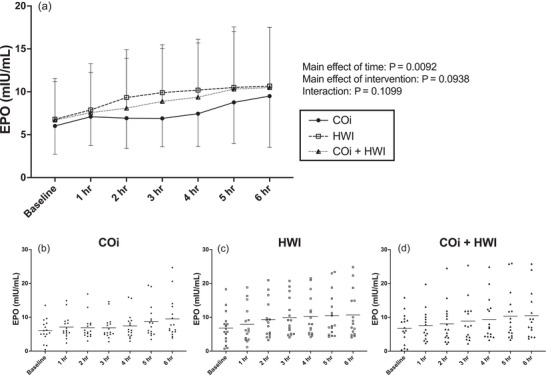
Comparing EPO concentration across the interventions. Data in (a) are presented as means ± SD. Data were analysed with two‐way mixed‐effects analysis with time and intervention as the two factors. *Significant pairwise comparisons (*P* < 0.05) compared to baseline within an intervention. Main effects of time and intervention, as well as interaction effects, are presented to the right of panel (a). COi is represented with filled circles and continuous lines; HWI is represented with open squares and dashed lines; COi + HWI is represented with half‐filled triangles and dotted lines. (a) EPO concentration over time during COi (*n* = 16), HWI (*n* = 16) and COi + HWI (*n* = 16) interventions. (b) Individual responses to COi. (c) Individual responses to HWI. (d) Individual responses to COi + HWI.

There was an effect of the study visit on baseline EPO concentration (*P* = 0.0419), but there were no pairwise differences when comparing baseline EPO concentrations on the first (5.78 ± 3.42 mIU/mL), second (6.15 ± 3.93 mIU/mL) and third (7.59 ± 4.98 mIU/mL) visits. EPO concentration increased over time in all three visits, and the pattern of this response did not differ between interventions (Figure 4a). Individual responses to each intervention are shown in Figure 4b–d.

### 3.4 Sex differences

**FIGURE 5:**
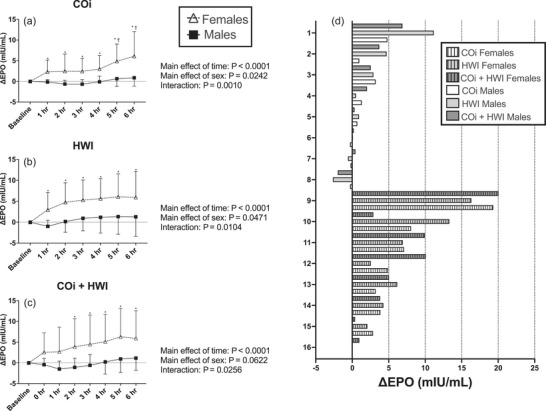
Sex differences in the change in EPO. Data in (a–c) are presented as means ± SD. Data were analysed with two‐way mixed‐effects analysis with time and sex as the two factors. Main effects of time and intervention, as well as interaction effects, are presented below each panel. *Significant pairwise comparisons (*P* < 0.05) compared to baseline within a sex; †significant pairwise comparisons (*P* < 0.05) comparing females to males within a time point. Females are represented with open triangles and dashed lines; males are represented with filled squares and continuous lines. (a) The change in EPO in females (*n* = 8) and males (*n* = 8) in response to COi; (b) the change in EPO in females (*n* = 8) and males (*n* = 8) in response to HWI; (c) the change in EPO in females (*n* = 8) and males (*n* = 8) in response to COi + HWI. For panel (d), maximum individual responses to each intervention are provided. COi is shown by white bars, HWI by light grey bars, COi + HWI by dark grey bars; males by solid bars, females by striped bars.

There was no effect of sex (*P* = 0.1118), nor an interaction between sex and study visit (*P* = 0.6983) on baseline EPO concentration. There was an interaction between sex and time on the change in EPO concentration for the COi (Figure 5a), HWI (Figure 5b) and COi + HWI (Figure 5c) interventions. Females increased EPO concentration on all three study visits while males did not (Figure 5). In addition, females had a significantly greater change in EPO concentration than males 5 and 6 h post‐COi (Figure 5a). Individual responses to each intervention are shown in Figure 5d.

Despite sex differences in the change in EPO concentration in response to all interventions, only the peak and change in HbCO were significantly different between females and males (Table 2). Females had a greater peak and change in HbCO in response to COi and COi + HWI interventions (Table 2). There were no differences between females and males in any stimulus data from the HWI intervention (Table 2).

**TABLE 2:** Sex differences in the stimulus for EPO secretion.

		Females	Males	*P*‐value
COi	Peak HbCO (%)	14.0 ± 2.4	11.7 ± 1.3	0.0300*
	∆ HbCO	12.5 ± 2.4	10.2 ± 1.1	0.0287*
	Min renal blood velocity (cm/s)	43.2 ± 5.0	43.1 ± 6.6	0.9679
	∆ Renal blood velocity	−3.4 ± 3.6	−3.2 ± 4.2	0.8957
	Max renal vascular resistance (cm/s/mmHg)	1.8 ± 0.3	1.7 ± 0.2	0.3329
	∆ Renal vascular resistance	−0.04 ± 0.17	−0.13 ± 0.21	0.4155
HWI	Min renal blood velocity (cm/s)	32.3 ± 6.6	32.5 ± 5.5	0.9646
	∆ Renal blood velocity	−13.5 ± 7.9	−14.1 ± 5.8	0.8582
	Max renal vascular resistance (cm/s/mmHg)	1.7 ± 0.4	1.7 ± 0.3	0.90503
	∆ Renal vascular resistance	0.02 ± 0.50	−0.13 ± 0.35	0.4981
	Max core temperature (°C)	38.1 ± 0.4	38.1 ± 0.3	0.6870
	∆ Core temperature (°C)	1.0 ± 0.4	1.2 ± 0.3	0.2283
	Max heart rate (bpm)	108 ± 13	96 ± 15	0.1057
	∆ Heart rate (bpm)	33 ± 7	37 ± 10	0.3552
COi + HWI	Peak HbCO (%)	14.0 ± 2.8	11.4 ± 1.1	0.0270*
	∆ HbCO	12.6 ± 2.8	9.9 ± 0.9	0.0181*
	Min renal blood velocity (cm/s)	36.8 ± 4.1	35.2 ± 8.0	0.6293
	∆ Renal blood velocity	−10.1 ± 7.1	−12.6 ± 5.9	0.4772
	Max renal vascular resistance (cm/s/mmHg)	1.5 ± 0.2	1.6 ± 0.2	0.1450
	∆ Renal vascular resistance	−0.3 ± 0.2	−0.2 ± 0.2	0.2614
	Max core temperature (°C)	38.2 ± 0.4	38.0 ± 0.3	0.2308
	∆ Core temperature (°C)	0.9 ± 0.6	1.3 ± 0.5	0.2832
	Max heart rate (bpm)	112 ± 18	100 ± 15	0.1811
	∆ Heart rate (bpm)	37 ± 8	37 ± 10	0.9554

*Note*: The mean ± standard deviation of the females and males is provided. Changes in stimulus values were calculated as the baseline value subtracted from the maximum or minimum value. *P*‐values represent comparisons between females and males. *n* = 8 females; *n* = 8 males. *P < 0.05.

## 4 DISCUSSION

In the present study, we found that COi and HWI, both independently and when combined, increased EPO concentration. However, contrary to our hypothesis, the combination of COi and HWI did not further augment the increased EPO concentrations above either COi or HWI alone. An additional important finding was that the males on average did not increase EPO while their female counterparts on average did. The reasons for this unexpected observation are unknown but are only partially explained by sex differences in the stimulus for EPO production. Additionally, it is intriguing that the males in the present study on average did not increase EPO concentration in response to COi, which contradicts several previous studies (Montero & Lundby, 2019; Schmidt et al., 2020; Wang et al., 2019). Reasons for these contradictory observations will be examined in greater detail in the subsequent subsections.

### 4.1 Effects of COi and HWI on EPO concentration

All three interventions significantly increased EPO concentration from baseline. This confirms results from other studies that show that COi alone can increase EPO concentration (Montero & Lundby, 2019; Schmidt et al., 2020; Wang et al., 2019). However, to the best of our knowledge, we are the first to show that heat alone can acutely increase EPO concentration in humans since other studies have shown no increase in EPO in response to heat stress (Akerman et al., 2017; Oberholzer et al., 2019). Despite previous research showing no change in EPO in response to whole‐body heating, the finding that both of our interventions increased EPO is expected given that we found that both interventions reduced at least one component of renal oxygen delivery, and this is presumably the mechanism causing increased EPO. However, we did not include a time‐control to measure EPO without the use of the three interventions because it has been confirmed that EPO does not acutely change over time after breathing normoxic gas (Montero & Lundby, 2019). Therefore, while extremely unlikely, we cannot confirm that the observed increase in EPO was not due to circadian fluctuations.

The finding that the interventions used in the present study increased EPO leads to the question of whether an increase in EPO of approximately 1–7 mIU/mL, which we observed in our study, is physiologically meaningful. To be physiologically meaningful, we are referring to a concentration of EPO that if sustained would elicit increases in Hb mass chronically. From the few studies that present both EPO and Hb mass data in response to COi, a small increase in EPO of approximately 2 mIU/mL over weeks was sufficient to elicit increases in Hb mass (Schmidt et al., 2020; Wang et al., 2019). While we are not aware of a physiologically significant effect of this EPO concentration acutely, this concentration is a substantial enough increase that when elevated chronically may elicit significant increases in red blood cell mass based on previous literature.

Contrary to our hypothesis, combining COi and HWI did not augment EPO secretion. This was unexpected given that COi and HWI presumably target different components of renal oxygen delivery. In the present study, we measured the supposed stimuli for EPO secretion during both HWI and COi and confirmed that COi only increased HbCO while HWI only reduced renal blood velocity. COi + HWI resulted in both increased HbCO and reduced renal blood velocity, so based on our stimulus data alone, this combined intervention theoretically should have augmented EPO secretion. Although previous studies suggest that COi administration may elicit an acute renal vasodilatation (Pauli et al., 1968), our data indicate that the COi administration in the present protocol did not have a vasodilatory effect in the kidneys given that there were no apparent differences in the magnitude of renal vasoconstriction during heat stress between HWI and COi + HWI, and that renal vascular resistance was unaltered by COi only. However, it is possible that we are not fully capturing the stimulus for EPO secretion and/or that EPO can be produced in response to a stimulus that is in addition to reductions in renal oxygen delivery.

One limitation to the present study is that Doppler ultrasound can only reliably measure renal blood velocity and not renal artery diameter, which is needed to calculate renal blood flow. It is generally assumed that the diameter of the renal artery does not change during acute interventions such as HWI because it is a conduit vessel (Chapman et al., 2020; Conboy et al., 2010), which has been confirmed in invasive human studies demonstrating that pharmacologically induced renal vasoconstriction causes decreases in renal artery blood velocity with no changes in renal artery diameter (Rocha et al., 2023). Rather, heat‐induced renal vasoconstriction is assumed to occur in downstream resistance vessels, so it is likely that the measured changes in renal artery blood velocity were a result of downstream vasoconstriction and not a result of changes in renal artery diameter. Nevertheless, we could not confirm that renal artery diameter was unchanged, which could have altered renal blood flow and the stimulus for EPO secretion. Similarly, we did not obtain arterial blood samples, so we were unable to directly measure $C_{aO_{2}}$ and instead were only able to measure HbCO to quantify the CO stimulus. We also did not measure variables such as renal metabolic rate, renal oxygen extraction, renal blood flow distribution, or oxygen utilization, which may have altered the true stimulus for EPO secretion. While it is possible that reduced renal perfusion is met with a compensatory increase in renal oxygen extraction, thereby preventing renal hypoxia, evidence from human and animal studies shows that reducing renal blood flow elicits renal hypoxia and does not result in altered oxygen extraction (Hess et al., 2022; Kim et al., 2021; Levy, 1960). Additionally, we were logistically unable to measure renal blood velocity throughout the 6 h post‐intervention time period, so it is possible that renal blood velocity throughout the day temporally correlated more strongly with changes in EPO concentrations. However, this is unlikely to contribute to our findings because it has been shown that renal blood velocity returns to baseline once skin temperature is restored to baseline (Chapman et al., 2019). Nevertheless, it is possible that the indirect measures obtained in the present study did not fully capture the stimulus for EPO secretion.

Additionally, to attempt to fully quantify the stimulus, we measured additional variables such as MAP and core temperature. An interesting observation is that the increase in core temperature does not temporally correlate with the reductions in renal blood velocity. Core temperature is unlikely to be the driving stimulus to reduce renal blood flow and is rather sympathetically mediated (Wilson, 2017) secondary to increased skin temperature (Minson et al., 1998). Because increases in core temperature likely do not drive reductions in renal blood velocity, this is unlikely to be the stimulus for EPO production.

While the kidney is the primary site of EPO production in the adult human, there are numerous extrarenal sites of EPO production. The liver is the primary site of EPO production during embryonic development and can still produce EPO in the adult human (Haase, 2010). In addition, EPO mRNA has been shown to be expressed in most other tissue types, including the brain, lungs, heart, bone marrow, spleen and reproductive tract (Haase, 2010). It is possible that we did not account for extrarenal stimuli and that extrarenal EPO production contributed to our findings.

Because COi + HWI did not augment EPO secretion beyond their individual effects, it is possible that hypoxia and heat have overlapping mechanisms of action, which could lead to them being antagonistic or hypo‐additive in their effects on EPO concentration rather than additive (Lloyd & Havenith, 2016). We chose COi and HWI because we wanted to provide stimuli that did not overlap in their mechanisms of action, but it is nevertheless possible that there is more overlap in how heat and hypoxia affect the kidney than previously understood. For example, higher core temperatures during HWI will right shift the oxyhaemoglobin dissociation curve, reducing arterial oxygen saturation and therefore content. However, the increase in core temperatures of only about 1°C at sea level is unlikely to cause a significant right shift and subsequent reduction in $S_{aO_{2}}$ to explain a large degree of overlap in the mechanisms of action of HWI and COi. Additionally, we confirmed that there was no effect of COi on renal blood velocity or renal vascular resistance, which was previously unknown. Therefore, if there are significant overlapping mechanisms of action between COi and HWI, they are currently unknown.

Although we are the first to examine this non‐augmented EPO concentration in response to COi + HWI, other studies have shown that heat and other forms of hypoxia do not provide additive stimuli, supporting the results from our study. One study found that EPO concentration was not significantly different in post‐exercise trials in which participants cycled in either heat and normobaric hypoxia ($F_{iO_{2}}$ of 14.5%) or heat only (Hayashi et al., 2020). Chronically, combining live‐high–train‐low with heat acclimation did not increase exercise performance beyond that achieved with heat acclimation training only (McCleave et al., 2017). Lastly, one study examined acute exercise performance decrements in response to hypoxia and heat and found that although combining heat and hypoxia worsened exercise performance below that of heat alone, combining heat and hypoxia did not reduce exercise performance below that of hypoxia alone (Bradbury et al., 2019). These studies, combined with the present data, indicate that heat and hypoxia likely do not provide an additive stimulus to augment EPO secretion, and future work should explore why.

### 4.2 Sex differences in EPO

We found that on average females increased EPO in response to each intervention while males did not. These results are partially explained by the stimulus data. Females had a higher peak and change in HbCO on both visits involving COi. Therefore, on the COi and COi + HWI visits, the higher HbCO in females likely provided a greater stimulus for EPO secretion. In our study design, we attempted to match the HbCO stimulus between sexes by administering a lower relative dose of CO to females to account for lower Hb mass. Nevertheless, the female participants had a greater HbCO stimulus. Future work should take caution in ensuring equal HbCO stimuli when examining sex differences.

However, this does not explain why there were sex differences in the change in EPO concentration during interventions involving HWI. There were no differences between females and males in any stimulus variable on the HWI visit that could explain the sex differences in EPO in response to HWI, so the reasons for the observed sex differences in EPO in response to HWI are unknown. However, as discussed above, it is possible the full stimulus for EPO production was not captured.

Additionally, it is important to discuss why we did not see an increase in EPO concentration in males in response to COi, whereas others have shown males increase EPO within 6 h post‐COi (Montero & Lundby, 2019; Wang et al., 2019). The discrepencies between these previous studies and the present study are most likely explained by the stimulus. Montero and Lundby (2019) raised HbCO to approximately 18% while the peak HbCO in the males in the present study averaged approximately 11.5%, indicating that the CO stimulus in the present study was less severe. While the CO stimulus provided by Wang et al. (2019) (HbCO of approximately 4.4%) was lower than in the present study, they also utilized exercise training immediately post‐COi, which could have augmented the stimulus. Additionally, EPO only increased approximately 1 mIU/mL (Wang et al., 2019), which is comparable to our study in which EPO increased approximately 1–2 mIU/mL in males in response to COi after 6 h.

Interestingly, in a meta‐analysis aimed to study variability in Hb mass increases in response to altitude training camps, males were found to respond better to altitude training camps, as measured by a greater increase in Hb mass (Nummela et al., 2021). Additionally, females and males similarly increased Hb mass in response to heat acclimation (Lundby et al., 2023). Those studies did not present EPO data, so it is unknown whether there were any differences in EPO concentrations between females and males. However, it is interesting to speculate that although males may have a blunted EPO secretion in response to acute stimuli compared to their female counterparts, functionally they may require a lower EPO concentration to significantly increase Hb mass. Despite females having greater increases in EPO concentrations in response to our acute interventions, chronic use of these interventions may not result in a greater increase in Hb mass in females compared to males. In support of this, in haemodialysis patients, females required a greater exogenous EPO dose to maintain Hb concentration in a healthy range compared to males (Coronado Daza & Cuchi, 2019). This supports the idea that a greater change in EPO or a greater concentration of EPO may be necessary to increase Hb mass in females. Therefore, there are possible differences between females and males in the translation from EPO to viable red blood cells, although this remains unstudied.

In addition to sex, it is worth considering other factors that could contribute to the heterogeneity of the participants in the present study. There may be differences in EPO due to factors such as race and/or birthplace that could effect EPO. There are known racial differences in EPO responsiveness, with Black haemodialysis patients requiring higher doses of exogenous EPO to maintain Hb mass (Lacson et al., 2008). Additionally, there may be a hyporesponsiveness in EPO production due to the high altitude birthplace. High‐altitude population studies show blunted responses to hypoxia in Tibetan and Ethiopian natives (Bigham, 2016; Petousi et al., 2014) given that these populations have long‐term generational adaptations as a result of high birth altitude. As noted in the results, one participant was born in a high‐altitude city in Colorado, and this female had the smallest change in EPO in response to all interventions. Additionally, the participant excluded for having EPO concentrations below the limits of detection at all time points was Black. Therefore, heterogeneity within our participant pool may explain some additional variability in EPO concentration.

### 4.3 Conclusions and ethical implications

Overall, this study shows that COi and HWI can increase EPO, although there is variability in who responds to these interventions. A significant observation was that there were no differences in EPO concentration between COi and HWI. Even though COi can increase EPO, it is nevertheless potentially dangerous, particularly if not used in well‐controlled environments. As others performing work in this field have also noted, there are ethical implications in the world of performance enhancement to using an intervention like COi (Laughlin, 2024). Our results emphasize that HWI is an equally effective alternative therapy that can be used by females wishing to increase circulating EPO concentration but without the risks or ethical implications associated with COi.

Our data also suggest that these interventions, at least acutely, are more effective in females. This may be partially due to an augmented stimulus in females in response to COi, but the reasons for the sex differences in EPO in response to HWI are still unknown. Therefore, future work should examine mechanisms underlying the sex differences in EPO.

## AUTHOR CONTRIBUTIONS

Kaitlyn G. DiMarco, Christopher L. Chapman, John R. Halliwill, Andrew T. Lovering, and Christopher T. Minson conceived and designed the work. All authors contributed to the acquisition, analysis, or interpretation of data for the work. Kaitlyn G. DiMarco drafted the work. All authors critically revised the work for important intellectual content. All authors approved the final version of the manuscript. All authors agree to be accountable for all aspects of the work in ensuring that questions related to the accuracy or integrity of any part of the work are appropriately investigated and resolved. All persons designated as authors qualify for authorship, and all those who qualify for authorship are listed.

## CONFLICT OF INTEREST

The authors declare no conflicts of interest.

## Data Availability

Data will be made available upon reasonable request from the corresponding author.
